# Impact of Depression on Hospitalization and Related Outcomes for Parkinson's Disease Patients: A Nationwide Inpatient Sample-Based Retrospective Study

**DOI:** 10.7759/cureus.1648

**Published:** 2017-09-03

**Authors:** Rikinkumar S Patel, Ramkrishna Makani, Zeeshan Mansuri, Upenkumar Patel, Rupak Desai, Amit Chopra

**Affiliations:** 1 Department of Psychiatry, Northwell Zucker Hillside Hospital; 2 Child and Adolescent Psychiatry, Children’s Hospital of Philadelphia; 3 Psychiatry, Texas Tech University Health Science Center; 4 Public Health, National University; 5 Research Coordinator, Atlanta Veterans Affairs Medical Center; 6 Psychiatry, Allegheny Health Network

**Keywords:** parkinson’s disease, depression, parkinsonism, mdd, deep brain stimulation, dbs, comorbidities

## Abstract

Background

Major Depressive Disorder (MDD) is a common comorbidity that significantly affects the quality of life and disease outcomes in Parkinson’s disease (PD) patients. No studies have been conducted to our knowledge to address the health care utilization and its outcomes in these patients. The aim of this study is to analyze and discern the differences in the hospitalization outcomes, comorbid conditions, and utilization of procedures in PD patients versus patients with comorbid MDD.

Methods

We used the Nationwide Inpatient Sample from the Healthcare Cost and Utilization Project from year’s 2010-2014. We identified PD and MDD as a primary and secondary diagnosis respectively using validated International Classification of Diseases, 9th Revision, and Clinical Modification codes. Pearson’s chi-square test and independent sample T-test were used for categorical data and continuous data, respectively. All statistical analysis was done by SPSS 22.0 in this study.

Results

Extensive analysis was performed on 63,912 patients with PD and 1445 patients with PD having MDD. Patients with comorbid depression had three times greater chances of disposition to acute care hospital (3.1% vs. 1.1%, p < 0.001). Median length of hospitalization was higher in Parkinson’s patients with depression (5.85 vs. 4.08 days; p < 0.001) though the median cost of hospitalization was low ($ 31,039 vs. $ 39,464; p < 0.001). This could be because therapeutic procedures performed during the hospitalization were lower in Parkinson’s patients with depression (0.53 vs. 0.89, p < 0.001). Utilization of Deep Brain Stimulation (DBS) was lower in Parkinson’s patients with depression (9.4% vs. 25.6%, p < 0.001). In­-hospital mortality was significantly higher in Parkinson’s patients with depression (1.4% vs. 1.1%; p < 0.001).

Conclusion

Our study establishes the negative impact of depression in PD with regards to hospitalization-related outcomes including the illness severity, comorbid conditions, risk of mortality, utilization of diagnostic and therapeutic procedures, the length of stay and disposition as compared to PD without depression.

## Introduction

Parkinson’s disease (PD) is a chronic, gradually progressive and fatal neurological disorder of the brain. The degeneration of Dopamine neurons in substantia nigra and deficiency of dopamine in the putamen (the regions involved in the regulation of movement) lead to cardinal clinical symptoms [1]. In the United States, the second most common neurodegenerative disease is PD with an estimated prevalence of 0.3% throughout the entire population [2]. The overall prevalence and global economic burden of PD in the world’s top 10 densely populated nations were investigated. It is estimated that the PD occurrence rate will double by 2030 [3]. One million people in America are affected by the PD and there are over 60,000 new cases reported every year [4-5].

Since there are no explicit tests to diagnose PD, it remains a clinical diagnosis based on the patient’s report and the combination of exhibited symptoms. The primary motor symptoms of PD include tremor, rigidity, akinesia or bradykinesia, and postural instability. The non-motor symptoms of PD include sleep disorders and psychiatric disorders including depression and anxiety. These non-motor symptoms have a huge influence that can negatively impact the health-related life quality of PD patients [6]. The severity of such symptoms indicates the progression of the disease, which results in higher costs of medical treatment for hospitalization and care coordination. Among the psychiatric disorders comorbid with PD, depression has the highest prevalence, followed by anxiety disorders, sleep disorders, and others [7]. Diagnosis of patients with PD is 1.89-4.26 times likely to get depression [8]. The severity of depression appears to correlate with disability and reduced quality of life in PD patients [9]. Depression seems to be underdiagnosed and undertreated in PD [7], even though adequate diagnosis and treatment can change the direction of the illness positively. Reduction of quality of life by depression in PD is independent of motor deficits. Therefore, depression should be treated independently of motor symptoms in patients with PD. Aspects of quality of life have been emphasized in diagnosis and treatment of PD and there appears to be relevant response criteria and goals in the pharmacological and non-pharmacological treatment of PD [10]. The impact of depression in PD patients ranges from mild manifested as an unwillingness to cooperate to a complete withdrawal and social isolation [11].

Previously studies were done to measure prevalence, neurobiology, and impact on health-related quality of life in PD patients with depression [12-14]. However, no previous studies were conducted to the best of our knowledge to address the health care utilization and hospitalization outcomes in PD patients with depression. Studies in the past evaluated medical and psychiatric comorbidities present in PD patients [15-17]. This study focussed on comorbidities that were present in PD with major depressive disorder (MDD) patients and compared its prevalence with PD patients without depression.

The main aims of this study are to analyze and discern the differences in the hospitalization outcomes, comorbid conditions and utilization of therapeutic and diagnostic procedures in PD patients versus PD patients with MDD. The results obtained from this study can be used effectively to assess the burden of care of MDD in PD and to develop strategies, and clinical care models to recognize and treat MDD to improve the quality of life of the patients by decreasing morbidity and mortality in PD.

## Materials and methods

Data source

A retrospective analysis was performed using the Healthcare Cost and Utilization Project's (HCUP) Nationwide Inpatient Sample (NIS) data from the years 2010 to 2014. The Agency for Healthcare Research and Quality (AHRQ) sponsors the HCUP databases that are specifically designed to determine and identify patterns in hospital utilization and cost across the United States. The HCUP-NIS database is the largest inpatient database available in the United States, which represents a sample of non-federal United States community hospitals. In 2010, there was an increase of 1,051 hospitals to 4,411 hospitals from 45 states in the United States that participated in NIS projects. More than seven million records of hospital stays were reported by these hospitals each year. A sample estimate of over 95% of discharges from the hospitals participated in NIS. We weighted the estimated samples to minimize the margin of error and to reflect all 50 states across the United States. The large sample size of the database enabled us to analyze rare conditions, uncommon treatments, and special patient populations. To protect the privacy of individual patients, physicians, and hospitals, the state and hospital identifiers are de-identified. There are many clinical and non-clinical hospitalization data elements recorded in the HCUP NIS database. Sample non-clinical information is patient’s demographic data, hospital characteristics (such as region and location), and total charges. Sample clinical-related information includes principal and secondary diagnosis, procedure codes (includes both ICD-9 and CCS codes), discharge status and the length of stay [18].

Variables of interest

Based on the ICD-9-CM diagnosis codes, we identified the individuals with a primary diagnosis of PD at the time of admission. Then, based on the ICD-9-CM diagnosis codes, the people with a primary diagnosis of PD and secondary diagnosis as MDD patients at the time of admission had been identified as the comparison group. In HCUP databases, more than 14,000 ICD-9-CM diagnosis codes and 3,900 procedure codes had been mentioned which were further classified and clustered into a lesser number of clinically appropriate categories by the AHRQ's Clinical Classification Software (CCS). This enabled the database to capture a large population of relatively similar conditions into a single group by making the information more useful for performing statistical analyses and developing reports. PD was identified using diagnosis code 332 and MDD was identified using diagnosis codes 296.2 and 296.3. To measure the differences in hospitalization outcomes in PD patients versus PD with MDD patients, the outcome variables of interest included the severity of illness that measures the loss of body functions, the risk of mortality that measures the likelihood of dying, disposition of patient and in-hospital mortality. In the NIS, we defined death as in-hospital mortality, and in this paper, it is reported as all-cause. We calculated the length-of-stay as the number of nights the patient remained in the hospital for a particular discharge. Length-of-stay in this analysis was all-cause. Total charges of hospitalization do not include professional fees and non-covered charges. If the source provided total charges with professional fees, then the professional fees were removed from the charge during HCUP processing. For the analysis, among the predictor variables, transfers out of the current hospital setting, a total number of ICD-9 CM procedures, Primary ICD-9 Procedures, and duration of the hospital stays were considered to be important. The primary and secondary diagnoses had been identified at the date of admission whereas we recorded comorbidities throughout the entire hospital stay. By a common definition, comorbidities were considered coexisting conditions with PD and PD with MDD, the index disorders under study. AHRQ comorbidity software was used to generate binary variables that identified seven comorbidities in discharge records using ICD-9-CM codes [19]. ICD-9 CM codes used for alcohol abuse are 291.0-291.3, 291.5, 291.8, 291.81, 281.82, 291.89, 291.9, 303.00-303.93 and 305.00-305.03; drug abuse are 292.0, 292.82-292.89, 292.9, 304.00-304.93, 305.20-305.93 and 648.30-648.34; psychosis are 295.00-298.9, 299.10 and 299.11; delirium are 290.11, 290.3, 290.41, 292.81, 293, 293.1, 292.11 and 292.12; and dementia are 290.0, 290.10-290.13, 290.20, 290.21, 290.3, 290.40-290.43, 294.0, 294.1, 331.0, 331.11, 331.19, 331.82, 331.89, 332.0 and 333.0.

Approaches

A retrospective analysis was performed over the HCUP-NIS database focusing on the determination of the hospital outcomes for PD and PD with MDD patients. Descriptive statistics were used to summarize the results. The mean and standard deviations were used to explain the continuous variables. Pearson’s chi-square test and independent sample T-test were used for categorical data and continuous data, respectively. On the other hand, the categorical variables were shown in percentage values. We used discharge weight which is given in NIS database to obtain national represent inpatient data. A p-value < 0.05 was used as a reference to determine the statistical significance test result. All statistical analysis was done by SPSS 22 in this study [20]. Our study database does not contain patient identification. Thus, we were not required to take Institution Review Board (IRB) permission for this study.

## Results

Extensive analysis was performed on 63,912 patients with PD and 1,445 patients with PD having MDD according to the hospital records. The preliminary results are presented below.

Demographics

Age Distribution

**Figure 1:**
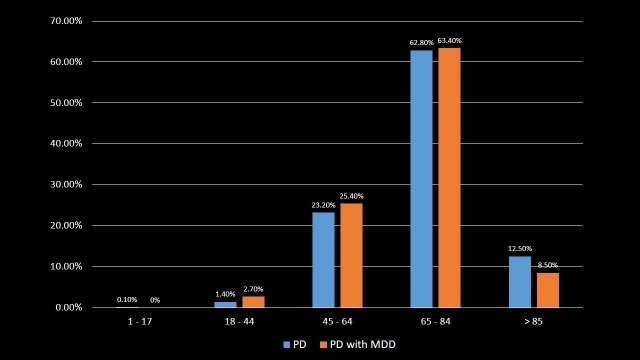
Distribution of Parkinson's disease and Parkinson's disease with major depressive disorder patients by the age group. PD: Parkinson's disease; MDD: Major depressive disorder.

The patients were distributed according to their age group as shown in Table 1 and Figure 1. The result indicates that a total of 62.8% of PD patients and 63.4% of PD with MDD patients were in the range of 65 to 84 years age group, comprising the most common age of presentation of PD and PD with MDD (p-value < 0.001). PD with MDD was less common in above 85 years age group which comprised of 8.5% of total PD with MDD patients in comparison to 12.5% of total PD patients (p-value < 0.001). The mean age of PD patients at the time of hospitalization was 71.7 years (S.D. = 11.296, p-value = 0.262) and 69.5 years (S.D. = 11.366, p-value = 0.262) for PD with MDD patients.

**Table 1:** Distribution of Parkinson's disease and Parkinson's disease with major depressive disorder patients by the age group. Significant p-values ≤ 0.05 at 95% confidence interval. PD: Parkinson's disease; MDD: Major depressive disorder.

Age (in years)	PD	PD with MDD	p-value
Mean Age ± S.D.	71.7 ± 11.296	69.5 ± 11.366	0.262
1-17	0.1%	0%	<0.001
18-44	1.4%	2.7%	<0.001
45-64	23.2%	25.4%	<0.001
65-84	62.8%	63.4%	<0.001
>85	12.5%	8.5%	<0.001

Sex Distribution

**Figure 2:**
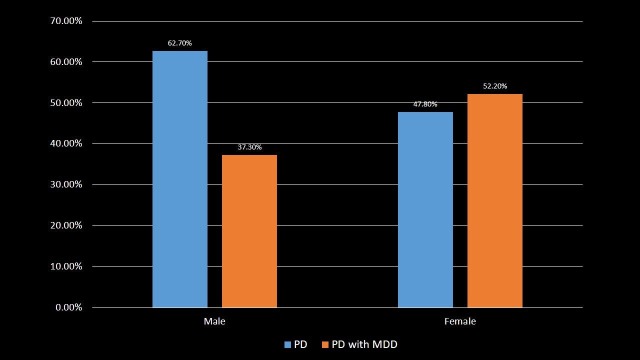
Distribution of Parkinson's disease and Parkinson's disease with major depressive disorder patients by the sex. PD: Parkinson's disease; MDD: Major depressive disorder.

The patients were distributed according to their sex as shown in Table 2 and Figure 2. PD was found in the greater proportion of males (62.7%), whereas PD with MDD was in the higher proportion of females (52.2%) (p-value < 0.001).

**Table 2:** Distribution of Parkinson's disease and Parkinson's disease with major depressive disorder patients by the sex. Significant p-values ≤ 0.05 at 95% confidence interval. PD: Parkinson's disease; MDD: Major depressive disorder.

Sex	PD	PD with MDD	p-value
Male	62.7%	37.3%	<0.001
Female	47.8%	52.2%	<0.001

Race Distribution

**Figure 3:**
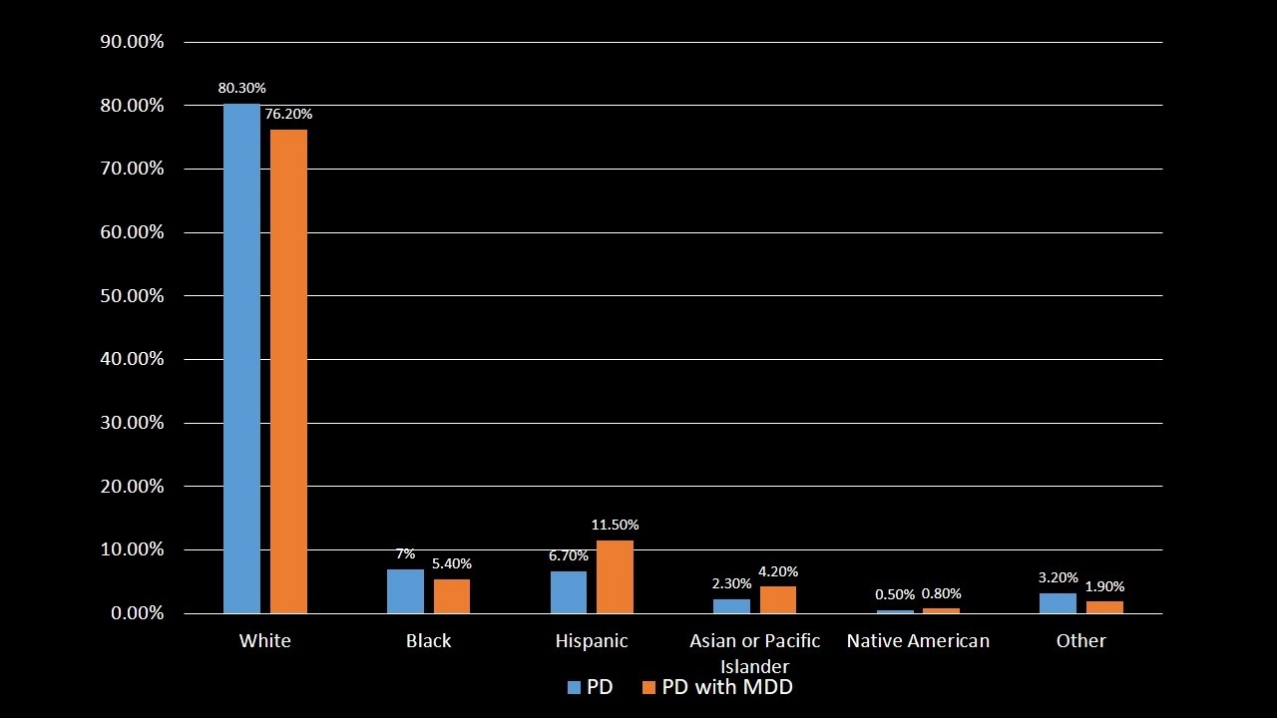
Distribution of Parkinson's disease and Parkinson's disease with major depressive disorder patients by the race. PD: Parkinson's disease; MDD: Major depressive disorder.

The patients were distributed according to their race as shown in Table 3 and Figure 3. PD was discovered in 80.3% of Whites and PD with MDD was found in 76.2% of Whites. The White race is an important risk factor for PD and PD with MDD. A greater proportion of PD with MDD was found in Hispanics (11.5%) and Asian or Pacific Islanders (4.2%) as compared to PD patients (6.7% and 2.3%, respectively) (p-value < 0.001).

**Table 3:** Distribution of Parkinson's disease and Parkinson's disease with major depressive disorder patients by the race. Significant p-values ≤ 0.05 at 95% confidence interval. PD: Parkinson's disease; MDD: Major depressive disorder.

Race	PD	PD with MDD	p-value
White	80.3%	76.2%	<0.001
Black	7%	5.4%	<0.001
Hispanic	6.7%	11.5%	<0.001
Asian or Pacific Islander	2.3%	4.2%	<0.001
Native American	0.5%	0.8%	<0.001
Other	3.2%	1.9%	<0.001

Geographic Region Distribution

**Figure 4:**
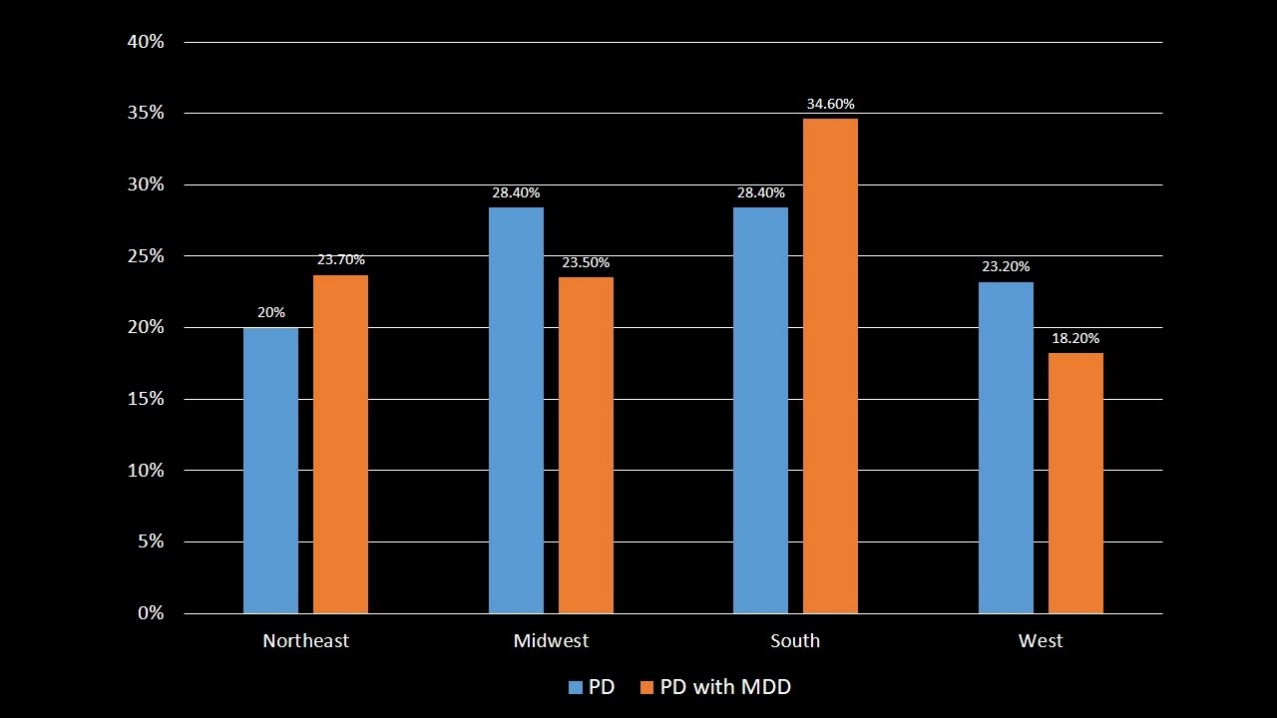
Distribution of Parkinson's disease and Parkinson's disease with major depressive disorder patients by the geographic region. PD: Parkinson's disease; MDD: Major depressive disorder.

The patients were distributed according to their geographic region as shown in Table 4 and Figure 4. The highest number of PD patients was found in Midwest (28.4%) and Southern (28.4%) regions of the United States, while the highest number of PD with MDD patients was found in Southern (34.6%) region of United States (p-value < 0.001). Prevalence of PD was lowest in Northeast region (20%), and PD with MDD was lowest in the Western region (18.2%) (p-value < 0.001).

**Table 4:** Distribution of Parkinson's disease and Parkinson's disease with major depressive disorder patients by the geographic region. Significant p-values ≤ 0.05 at 95% confidence interval. PD: Parkinson's disease; MDD: Major depressive disorder.

Region	PD	PD with MDD	p-value
Northeast	20%	23.7%	<0.001
Midwest	28.4%	23.5%	<0.001
South	28.4%	34.6%	<0.001
West	23.2%	18.2%	<0.001

Disposition/transfer of patient

**Figure 5:**
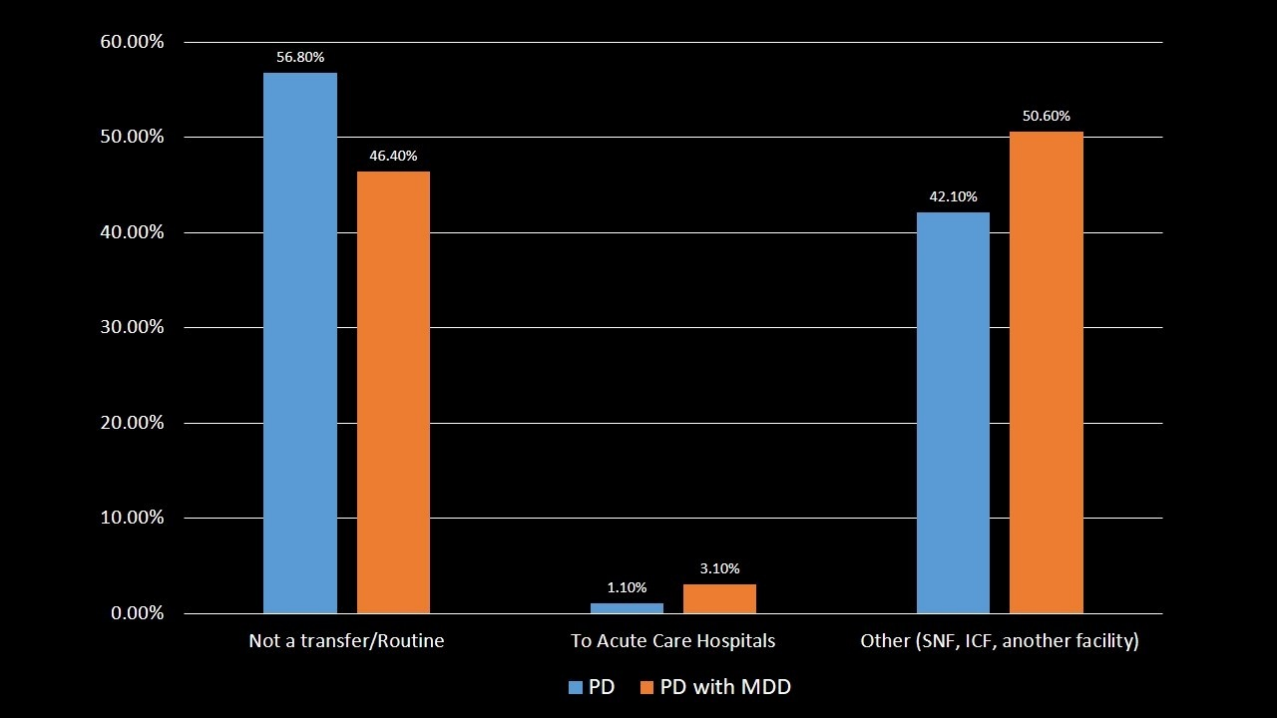
Distribution of Parkinson's disease and Parkinson's disease with major depressive disorder patients by the transfer/disposition. PD: Parkinson's disease; MDD: Major depressive disorder; SNF: Skilled nursing facility; INF: Intermediate nursing facility.

The patients were distributed according to transfer/disposition as shown in Table 5 and Figure 5. A major proportion of PD with MDD patients (50.6%) and PD patients (42.1%) was transferred to skilled nursing facility (SNF), intermediate nursing facility (INF) or another type of facility. PD with MDD patients had three times greater chances of disposition to short-term or acute care hospital, as 3.1% PD with MDD patients were transferred to such facilities in comparison to 1.1% PD patients (p-value < 0.001). This indicates that greater numbers of PD with MDD patients needed a specialized care in comparison to PD patients.

**Table 5:** Distribution of Parkinson's disease and Parkinson's disease with major depressive disorder patients by the transfer/disposition. Significant p-values ≤ 0.05 at 95% confidence interval. PD: Parkinson's disease; MDD: Major depressive disorder; SNF: Skilled nursing facility; INF: Intermediate nursing facility.

Disposition of patient	PD	PD with MDD	p-value
Not a transfer/routine	56.8%	46.4%	<0.001
To acute care hospitals	1.1%	3.1%	<0.001
Other (SNF, INF, another facility)	42.1%	50.6%	<0.001

Risk of mortality

**Figure 6:**
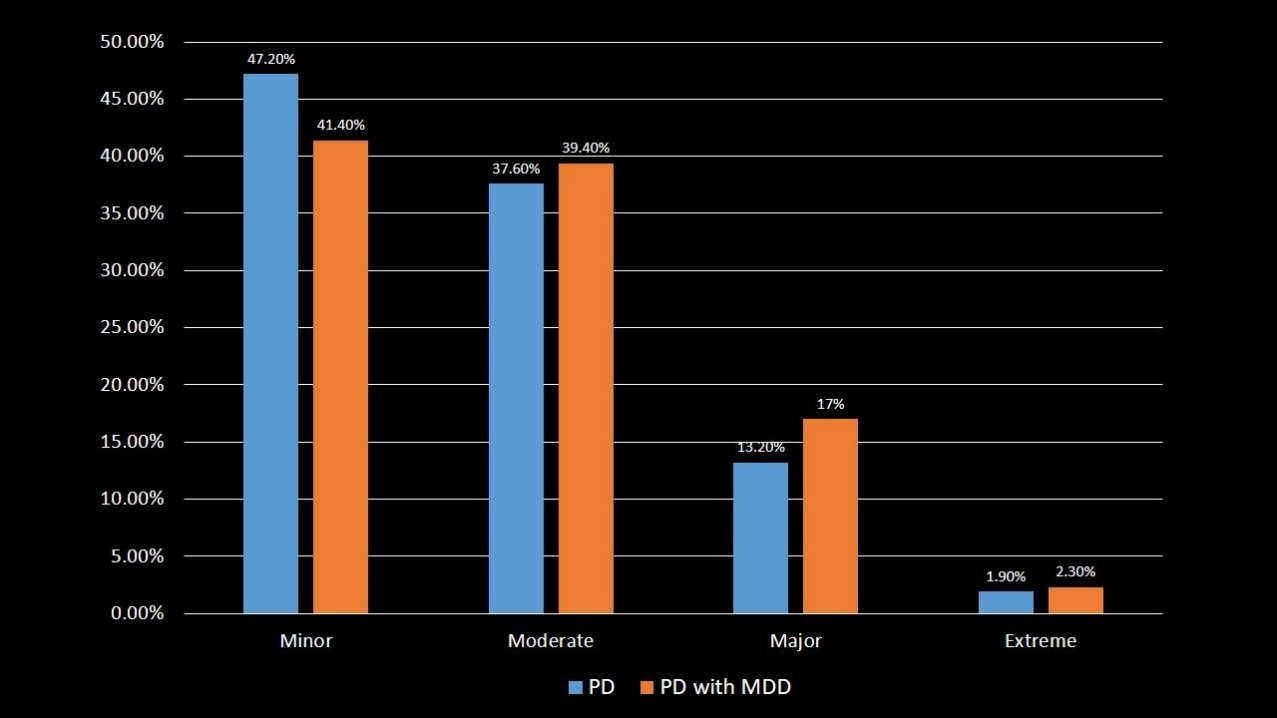
Risk of mortality in Parkinson's disease and Parkinson's disease with major depressive disorder patients. PD: Parkinson's disease; MDD: Major depressive disorder.

About 17% and 2.3% of PD with MDD patients had a major and extreme probability of dying respectively, which was higher as compared to 13.2% and 1.9% of PD patients with the risk of major and extreme probability of dying, respectively (p-value < 0.001). This indicates that PD with MDD patients are at higher risk of in-hospital mortality as compared to PD patients. The patients were distributed according to the risk of mortality in Table 6 and Figure 6.

**Table 6:** Risk of mortality in Parkinson's disease and Parkinson's disease with major depressive disorder patients. Significant p-values ≤ 0.05 at 95% confidence interval. PD: Parkinson's disease; MDD: Major depressive disorder.

Risk of mortality	PD	PD with MDD	p-value
Minor	47.2%	41.4%	<0.001
Moderate	37.6%	39.4%	<0.001
Major	13.2%	17%	<0.001
Extreme	1.9%	2.3%	<0.001

Severity of illness

**Figure 7:**
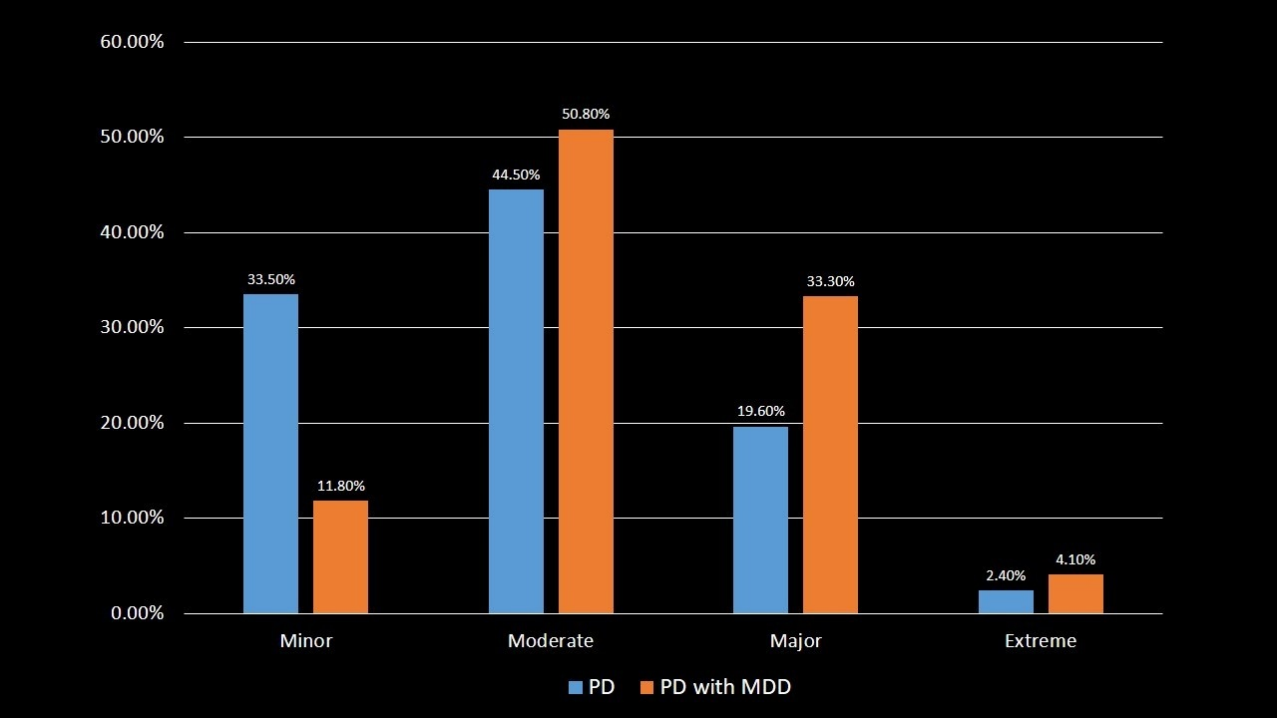
Severity of illness in Parkinson's disease and Parkinson's disease with major depressive disorder patients. PD: Parkinson's disease; MDD: Major depressive disorder.

About 33.3% and 4.1% of PD with MDD patients had a risk of major and extreme loss of function respectively, which was higher as compared to 19.6% and 2.4% of PD patients with a risk of major and extreme loss of function respectively (p-value < 0.001). This indicates that PD with MDD patients have about twice greater risk of major or extreme loss of function as compared to PD patients. The patients were distributed according to the severity of illness in Table 7 and Figure 7.

**Table 7:** Severity of illness in Parkinson's disease and Parkinson's disease with major depressive disorder patients. Significant p-values ≤ 0.05 at 95% confidence interval. PD: Parkinson's disease; MDD: Major depressive disorder.

Severity of illness	PD	PD with MDD	p-value
Minor	33.5%	11.8%	<0.001
Moderate	44.5%	50.8%	<0.001
Major	19.6%	33.3%	<0.001
Extreme	2.4%	4.1%	<0.001

Length of stay

The average duration of stay for PD with MDD patients was 5.85 days (S.D. = 7.323; p-value < 0.001), which was higher as compared to PD patients who had the average length of stay for 4.08 days (S.D. = 8.511; p-value < 0.001).

Total charges of hospitalization

Average total charges of hospitalization of PD with MDD patients were $ 31,038.99 (S.D. = 32537.986; p-value < 0.001) which was lower as compared to the $ 39,463.89 (S.D. = 43656.466; p-value < 0.001) average total charges for hospitalization of PD patients. PD with MDD patients had lower hospitalization expense as compared to PD patients despite having a longer duration of stay in the hospital. This could be due to less number of therapeutic procedures utilized in PD with MDD patients which is discussed in later part of this section.

In-hospital mortality

The risk of in-hospital mortality was higher in PD with MDD patients as compared to PD patients without depression. A total of 1.4% PD with MDD patients died in the hospital at the time of recent admission, which raised the in-hospital mortality of these patients as compared to 1.1% PD patients who passed away in the hospital (p-value < 0.001).

Procedures

**Figure 8:**
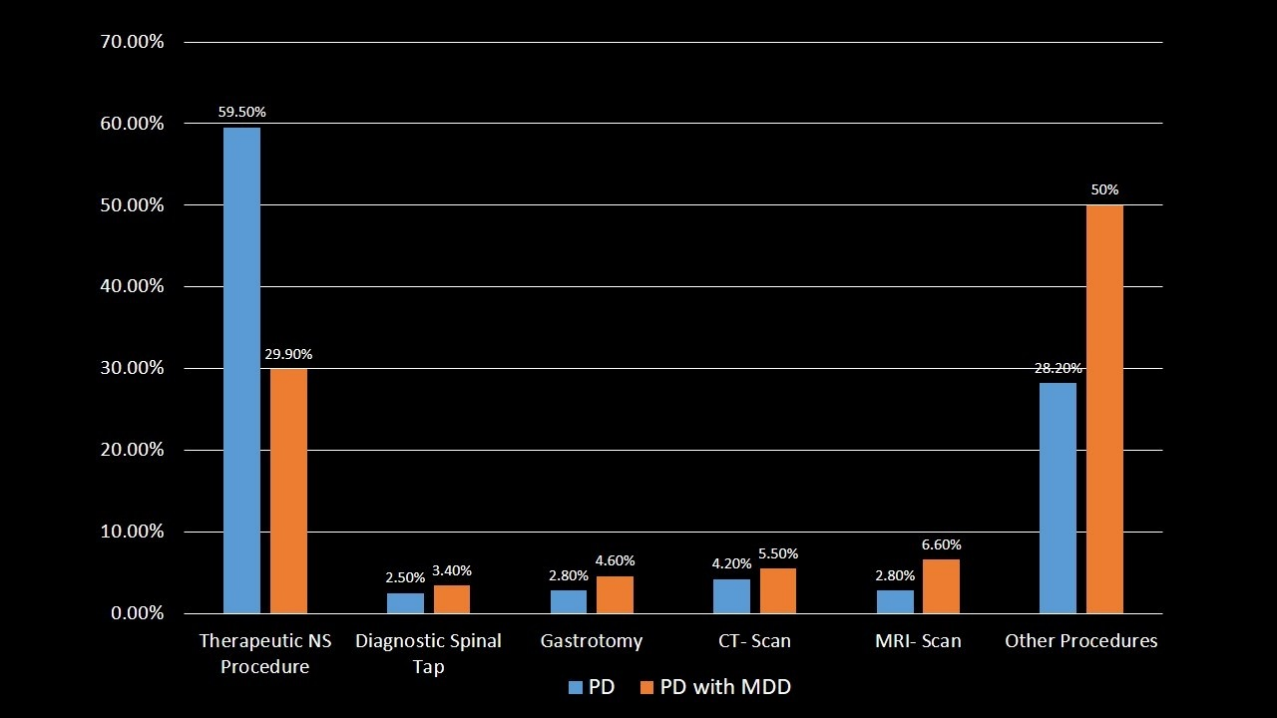
Procedures in Parkinson's disease and Parkinson's disease with major depressive disorder patients. PD: Parkinson's disease; MDD: Major depressive disorder; NS: Nervous system; CT: Computed tomography; MRI: Magnetic resonance imaging.

Average number of procedures performed during the hospitalization was higher in PD patients, i.e., 0.89 (S.D. = 1.309, p-value < 0.001) compared to 0.53 procedures in PD with MDD patients (S.D. = 0.977, p-value < 0.001). A total of 29,343 (45.9%) PD patients and 437 (30.2%) PD with MDD patients underwent therapeutic or diagnostic procedures during the hospitalization. The most common procedure performed was Therapeutic Nervous system procedure, i.e., 59.5% in PD patients, but lower in PD with MDD patients (29.9%). Diagnostic procedures like computed tomography (CT)-scan Head, magnetic resonance imaging (MRI) scan and diagnostic spinal tap were performed more in PD with MDD patients (5.5%, 6.6% and 3.4%, respectively) compared to PD patients (4.2%, 2.8% and 2.5%, respectively) (p-value < 0.001). Invasive procedures like Gastrotomy (temporary or permanent placement) were performed 1.5 times greater in PD with MDD patients as compared to PD patients (p-value < 0.001). The patients were distributed according to the utilization of procedures in Table 8 and Figure 8.

**Table 8:** Procedures in Parkinson's disease and Parkinson's disease with major depressive disorder patients. Significant p-values ≤ 0.05 at 95% confidence interval. PD: Parkinson's disease; MDD: Major depressive disorder; CT: Computed tomography; MRI: Magnetic resonance imaging.

Procedure	PD	PD with MDD	p-value
Therapeutic nervous system procedure	59.5%	29.9%	<0.001
Diagnostic spinal tap	2.5%	3.4%	<0.001
Gastrotomy	2.8%	4.6%	<0.001
CT-scan	4.2%	5.5%	<0.001
MRI-scan	2.8%	6.6%	<0.001
Other procedures	28.2%	50%	<0.001

**Figure 9:**
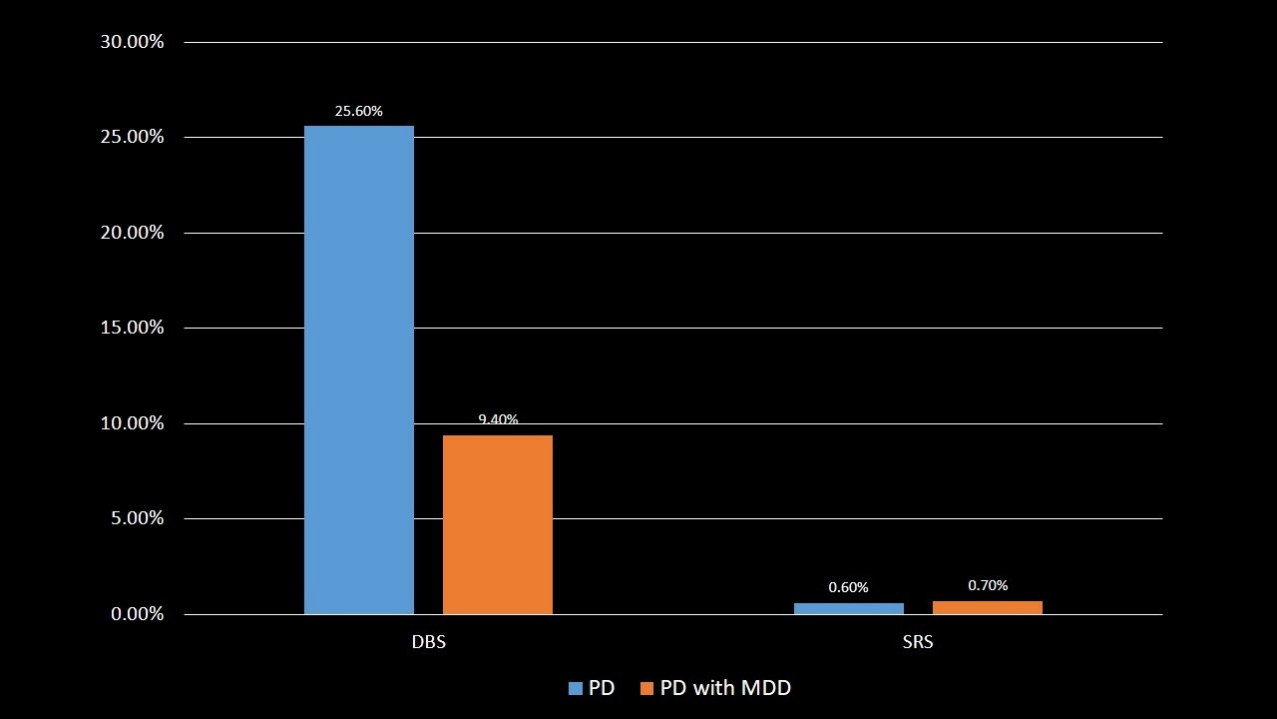
Utilization of deep brain stimulation and stereotactic radiosurgery in Parkinson's disease and Parkinson's disease with major depressive disorder patients. PD: Parkinson's disease; MDD: Major depressive disorder; DBS: Deep brain stimulation; SRS: Stereotactic radiosurgery.

The most important therapeutic procedure used during hospitalization is deep brain stimulation (DBS) in which there were 136, i.e., 9.4% of total PD with MDD patients, which is very low as compared to the use of DBS in 16,368, i.e., 25.6% of PD patients (p-value < 0.001). On the contrary, only 10 (0.7%) PD with MDD patients and 396 (0.6%) PD patients were treated with stereotactic radiosurgery (SRS) during the hospitalization, though this result was not statistically significant (p-value = 0.728). The patients were distributed according to the utilization of DBS and SRS procedures in Table 9 and Figure 9.

**Table 9:** Utilization of deep brain stimulation and stereotactic radiosurgery in Parkinson's disease and Parkinson's disease with major depressive disorder patients. Significant p-values ≤ 0.05 at 95% confidence interval. PD: Parkinson's disease; MDD: Major depressive disorder; DBS: Deep brain stimulation; SRS: Stereotactic radiosurgery.

Procedure	PD	PD with MDD	p-value
DBS	25.6%	9.4%	<0.001
SRS	0.6%	0.7%	0.728

Comorbidities

Comorbidities present in PD with MDD patients are alcohol abuse (3.7%), psychosis (100%) and drug abuse (5%) compared to 1.4%, 0%, and 0.8% respectively in PD patients (p-value < 0.001). Thus, psychosis was noted to be highly prevalent comorbidity in PD with MDD patients. Dementia was seen in 10.2% of PD and 11.5% of PD with MDD patients (p-value = 0.059). Delirium comorbidity was not present in PD patients but was seen in 0.3% of PD with MDD patients (p-value < 0.001).

## Discussion

This analysis of population-based hospital data from patients with PD and PD with MDD reveals the impact of depression on the hospitalization and related outcomes in PD. Prevalence rates of depressive syndromes in PD that are reported in the literature vary widely, ranging from 2.7% to more than 90% [21-22]. Possible reasons for this variation include the nature of the population studied, the way the diagnosis is established, the types of depressive disorders included in the study, and the statistical measures used. Our study had analyzed NIS data with hospitalization from 2010 to 2014 and over a five-year period, 2.26% of patients with PD had a diagnosis of MDD. Patients with younger age at the onset appear to have higher rates of depression than those with later age at the onset [23]. Our study results supported the earlier observations, as the higher proportion of PD with MDD patients were present compared to PD patients in 45-64 years and 65-84 years age group as compared to >85 years age group. In general population, women have consistently been shown to have a two-fold chance to develop a depressive disorder compared to men [24]. PD with MDD was seen in greater proportion in female patients which supports the fact that female gender is a risk factor for depression in PD.

Depression in PD is associated with increased disability, worse health-related quality of life and higher caregiver burden [25-26]. PD with MDD patients were transferred more to SNF, INF or another type of facility as compared to PD patients. Also, PD with MDD patients had three times greater chances of disposition to short-term or acute care hospital that may be due to worsened health outcomes. PD with MDD patients had twice the risk of severity of illness and loss of body function compared to PD patients. This likely led to an increased likelihood of dying in PD with MDD patients which indeed was associated with higher in-hospital mortality compared to PD patients.

Psychosis comorbidity was observed in higher proportion of PD with MDD patients followed by other comorbidities like alcohol abuse, drug abuse, and delirium, as compared to PD patients without depression. The presence of psychosis is a strong predictor of MDD in PD patients [27]. So our study supports the observations made earlier. Psychosis and delirium comorbidity was present only in PD with MDD patients and absent in PD patients without depression. Dementia was seen in a greater proportion of PD with MDD patients compared to PD patients without depression. The presence of comorbidities and its treatment procedures had significantly reduced the quality of life for PD patients diagnosed with MDD.

Therapeutic procedures were less commonly performed in PD with MDD patients in comparison to PD patients, whereas diagnostic procedures like CT-scan Head, MRI scan and Diagnostic Spinal Tap, and invasive procedures like Gastrotomy were performed in greater proportion in PD with MDD patients. DBS, which is an important treatment option for PD with dyskinesia and motor complications, was about three times less commonly used in PD with MDD patients as compared to PD patients. The lack of consideration for DBS in PD patients with MDD is concerning as these patients are likely to equally do well in terms of the motor and non-motor symptoms in a prospective study, especially if provided with multidisciplinary care including pre- and post-DBS psychiatric follow-up [28]. Overall, PD with MDD patients had a greater median length of stay in the hospital with higher in-hospital mortality and lower total cost of hospitalization when compared to PD patients.

The biggest strength of our study lies in the national representation of the dataset, with a uniform collection of data, through ICD-9-CM codes over five years. This is the first study, to our knowledge, to report the impact of MDD in PD patients regarding hospital outcomes, morbidity, mortality, and utilization of therapeutic procedures. Using the NIS dataset, we got a large sample size as we included 63,912 PD patients and 1445 PD patients with MDD. This dataset is subject to minimal reporting bias, and all information is coded independently of the individual practitioner, making it a more reliable source.

However, our study has several limitations. The NIS is an administrative database and lacks the patient level data needed to make accurate clinical associations. Further, such retrospective studies are always subject to selection bias, which might be accentuated by the moderate sensitivity of diagnostic codes for PD and MDD. We could not account for re-hospitalizations, given the nature of the database, although they add to the total inpatient burden. Despite these potential limitations, the NIS database provides an unparalleled population-based perspective on disease associations with systematic and temporal factors and provides a rationale for further in-depth studies.

## Conclusions

Our study establishes the negative impact of depression in PD in terms of hospitalization-related outcomes including the illness severity, comorbid conditions, risk of mortality, utilization of diagnostic and therapeutic procedures, length of stay and disposition as compared to PD alone. Further research to guide the development of clinical care models for targeting identification and treatment of depression in PD is warranted to both reduce mortality and morbidity and improve quality of care in PD with MDD.
